# Supplementary material for: Hot spot prediction in protein-protein interactions by an ensemble system
Source: BMC Syst Biol. 2018 Dec 31;12(Suppl 9):132. doi: 10.1186/s12918-018-0665-8 (PMC6311905; doi:10.1186/s12918-018-0665-8)
Supplement: Supplementary file 2 — All relevant MATLAB code. A simple Matlab implement of our predictor. (ZIP 19,742 kb) [file 12918_2018_665_MOESM2_ESM.zip › Additional file 2/Read ASA/ASA website/dbMPIKT/4RA0C.html]

 NetSurfP - Protein Surface Accessibility and Secondary Structure Predictions

  

|  |  |  |
| --- | --- | --- |
|  |  | NetSurfP - Protein Surface Accessibility and Secondary Structure Predictions  Technical University of Denmark |

  


---

```
# For publication of results, please cite:
# A generic method for assignment of reliability scores applied to solvent accessibility predictions.
# Bent Petersen, Thomas Nordahl Petersen, Pernille Andersen, Morten Nielsen and Claus Lundegaard
# BMC Structural Biology 2009, 9:51 doi:10.1186/1472-6807-9-51
#
# Column 1: Class assignment - B for buried or E for Exposed - Threshold: 25% exposure, but not based on RSA
# Column 2: Amino acid
# Column 3: Sequence name
# Column 4: Amino acid number
# Column 5: Relative Surface Accessibility - RSA
# Column 6: Absolute Surface Accessibility
# Column 7: Z-fit score for RSA prediction
# Column 8: Probability for Alpha-Helix
# Column 9: Probability for Beta-strand
# Column 10: Probability for Coil
E E  4RA0C                  1    0.861 150.329   1.324   0.003   0.003   0.994
E E  4RA0C                  2    0.686 119.897   1.301   0.005   0.015   0.979
B S  4RA0C                  3    0.111  13.033  -1.203   0.004   0.085   0.910
E P  4RA0C                  4    0.547  77.563   1.388   0.004   0.514   0.481
B F  4RA0C                  5    0.155  31.088  -0.791   0.002   0.816   0.182
E V  4RA0C                  6    0.399  61.403   0.422   0.002   0.816   0.182
E S  4RA0C                  7    0.420  49.177   0.542   0.003   0.718   0.279
E N  4RA0C                  8    0.538  78.836   0.815   0.005   0.336   0.660
B P  4RA0C                  9    0.204  28.976  -0.393   0.005   0.045   0.951
E G  4RA0C                 10    0.621  48.896   0.640   0.004   0.085   0.910
E N  4RA0C                 11    0.603  88.279   0.021   0.005   0.336   0.660
B I  4RA0C                 12    0.201  37.259   1.376   0.002   0.816   0.182
E T  4RA0C                 13    0.454  62.914   1.286   0.001   0.900   0.099
B G  4RA0C                 14    0.055   4.360   0.484   0.001   0.900   0.099
E A  4RA0C                 15    0.453  49.877   1.089   0.002   0.816   0.182
E R  4RA0C                 16    0.456 104.355   0.700   0.004   0.197   0.799
E G  4RA0C                 17    0.684  53.839  -0.560   0.005   0.015   0.979
E L  4RA0C                 18    0.506  92.612   1.161   0.005   0.045   0.951
E T  4RA0C                 19    0.543  75.286   1.329   0.004   0.514   0.481
B G  4RA0C                 20    0.039   3.093   0.069   0.001   0.900   0.099
E T  4RA0C                 21    0.371  51.416   2.209   0.001   0.959   0.040
B L  4RA0C                 22    0.037   6.738   0.367   0.001   0.959   0.040
E R  4RA0C                 23    0.343  78.524   1.681   0.001   0.959   0.040
B C  4RA0C                 24    0.016   2.303   1.164   0.001   0.900   0.099
E Q  4RA0C                 25    0.391  69.815   1.849   0.001   0.959   0.040
B L  4RA0C                 26    0.056  10.327  -0.304   0.001   0.959   0.040
E Q  4RA0C                 27    0.495  88.478   0.389   0.003   0.718   0.279
E V  4RA0C                 28    0.608  93.403  -1.987   0.004   0.197   0.799
E Q  4RA0C                 29    0.600 107.071  -2.344   0.005   0.015   0.979
E G  4RA0C                 30    0.520  40.900  -0.985   0.005   0.015   0.979
E E  4RA0C                 31    0.333  58.140  -0.465   0.004   0.085   0.910
E P  4RA0C                 32    0.484  68.651   0.397   0.005   0.262   0.733
B P  4RA0C                 33    0.068   9.706  -0.029   0.004   0.616   0.381
E E  4RA0C                 34    0.332  58.070   1.281   0.001   0.900   0.099
B V  4RA0C                 35    0.031   4.703   0.409   0.001   0.959   0.040
B H  4RA0C                 36    0.213  38.727   1.608   0.001   0.959   0.040
B W  4RA0C                 37    0.029   7.071   0.536   0.001   0.959   0.040
B L  4RA0C                 38    0.241  44.054   1.479   0.001   0.959   0.040
B R  4RA0C                 39    0.181  41.564   0.803   0.002   0.816   0.182
E D  4RA0C                 40    0.571  82.324  -0.407   0.005   0.015   0.979
E G  4RA0C                 41    0.731  57.545  -1.577   0.016   0.005   0.979
E Q  4RA0C                 42    0.511  91.282   0.198   0.004   0.616   0.381
E I  4RA0C                 43    0.513  94.850   0.548   0.003   0.718   0.279
B L  4RA0C                 44    0.071  12.927  -0.328   0.022   0.552   0.426
E E  4RA0C                 45    0.465  81.253   0.294   0.022   0.359   0.619
E L  4RA0C                 46    0.465  85.196  -1.463   0.019   0.141   0.840
E A  4RA0C                 47    0.555  61.117  -1.863   0.018   0.047   0.935
E D  4RA0C                 48    0.518  74.615  -1.894   0.018   0.088   0.893
E S  4RA0C                 49    0.466  54.650  -0.981   0.021   0.451   0.528
E T  4RA0C                 50    0.333  46.229  -0.685   0.023   0.655   0.322
E Q  4RA0C                 51    0.329  58.706  -0.444   0.018   0.846   0.136
B T  4RA0C                 52    0.239  33.205  -1.008   0.018   0.846   0.136
E Q  4RA0C                 53    0.309  55.187  -1.187   0.018   0.846   0.136
B V  4RA0C                 54    0.304  46.740  -1.217   0.023   0.655   0.322
E P  4RA0C                 55    0.446  63.259  -1.368   0.022   0.359   0.619
E L  4RA0C                 56    0.475  86.918  -1.950   0.020   0.205   0.775
E G  4RA0C                 57    0.547  43.041  -1.916   0.056   0.142   0.802
E E  4RA0C                 58    0.498  87.001  -1.881   0.064   0.216   0.721
E D  4RA0C                 59    0.428  61.660  -1.813   0.064   0.216   0.721
E E  4RA0C                 60    0.459  80.240  -1.438   0.022   0.359   0.619
E Q  4RA0C                 61    0.409  72.994  -0.970   0.022   0.552   0.426
E G  4RA0C                 62    0.339  26.656  -0.969   0.023   0.655   0.322
E D  4RA0C                 63    0.393  56.602  -1.132   0.018   0.846   0.136
E W  4RA0C                 64    0.341  81.986  -1.034   0.002   0.816   0.182
E I  4RA0C                 65    0.361  66.859  -1.270   0.004   0.616   0.381
E V  4RA0C                 66    0.499  76.712  -1.596   0.005   0.262   0.733
E A  4RA0C                 67    0.493  54.351  -0.830   0.005   0.262   0.733
B S  4RA0C                 68    0.153  17.967  -0.338   0.004   0.514   0.481
B Q  4RA0C                 69    0.246  44.007   0.308   0.002   0.816   0.182
B L  4RA0C                 70    0.025   4.559   0.612   0.001   0.900   0.099
E R  4RA0C                 71    0.268  61.372   1.552   0.001   0.959   0.040
B I  4RA0C                 72    0.042   7.751   0.384   0.001   0.900   0.099
E T  4RA0C                 73    0.476  66.063   1.528   0.002   0.816   0.182
E S  4RA0C                 74    0.508  59.584   0.825   0.004   0.616   0.381
B L  4RA0C                 75    0.065  11.938   0.138   0.004   0.420   0.576
E Q  4RA0C                 76    0.456  81.495   1.197   0.005   0.336   0.660
E L  4RA0C                 77    0.566 103.616   0.622   0.005   0.262   0.733
E S  4RA0C                 78    0.658  77.059   0.315   0.004   0.138   0.858
B D  4RA0C                 79    0.255  36.789  -0.775   0.004   0.085   0.910
E T  4RA0C                 80    0.548  76.063   0.103   0.004   0.138   0.858
B G  4RA0C                 81    0.165  12.978  -1.823   0.003   0.718   0.279
E Q  4RA0C                 82    0.443  79.066   1.900   0.001   0.900   0.099
B Y  4RA0C                 83    0.049  10.535   0.877   0.001   0.959   0.040
E Q  4RA0C                 84    0.269  48.043   2.077   0.001   0.959   0.040
B C  4RA0C                 85    0.014   1.994   1.604   0.001   0.959   0.040
E L  4RA0C                 86    0.265  48.521   2.019   0.001   0.959   0.040
B V  4RA0C                 87    0.029   4.473  -0.013   0.001   0.959   0.040
E F  4RA0C                 88    0.437  87.746   0.781   0.001   0.959   0.040
B L  4RA0C                 89    0.093  17.083  -0.427   0.004   0.514   0.481
E G  4RA0C                 90    0.618  48.637  -0.337   0.004   0.085   0.910
E H  4RA0C                 91    0.486  88.367  -0.963   0.004   0.085   0.910
E Q  4RA0C                 92    0.473  84.496  -1.190   0.004   0.138   0.858
E T  4RA0C                 93    0.569  78.948  -0.506   0.005   0.336   0.660
E F  4RA0C                 94    0.406  81.384  -0.724   0.004   0.616   0.381
E V  4RA0C                 95    0.439  67.490  -0.044   0.003   0.718   0.279
E S  4RA0C                 96    0.371  43.505  -0.340   0.002   0.816   0.182
E Q  4RA0C                 97    0.349  62.260   0.811   0.001   0.900   0.099
E P  4RA0C                 98    0.400  56.788   1.117   0.001   0.959   0.040
B G  4RA0C                 99    0.048   3.778   0.144   0.001   0.959   0.040
E Y  4RA0C                100    0.322  68.769   1.606   0.001   0.959   0.040
B V  4RA0C                101    0.032   4.857   0.517   0.001   0.959   0.040
E R  4RA0C                102    0.299  68.357   1.657   0.001   0.959   0.040
B L  4RA0C                103    0.042   7.745  -0.018   0.001   0.959   0.040
E E  4RA0C                104    0.377  65.879   0.636   0.001   0.900   0.099
B G  4RA0C                105    0.244  19.171   0.280   0.004   0.616   0.381
E L  4RA0C                106    0.314  57.512   0.823   0.004   0.085   0.910
B P  4RA0C                107    0.131  18.532  -0.472   0.004   0.197   0.799
E Y  4RA0C                108    0.464  99.178   1.099   0.002   0.816   0.182
B F  4RA0C                109    0.085  17.080   0.177   0.001   0.900   0.099
E L  4RA0C                110    0.333  60.936   0.346   0.001   0.900   0.099
E E  4RA0C                111    0.440  76.885   0.132   0.003   0.718   0.279
E E  4RA0C                112    0.438  76.431   0.600   0.005   0.262   0.733
E P  4RA0C                113    0.343  48.700  -0.962   0.005   0.045   0.951
E E  4RA0C                114    0.686 119.862   0.117   0.004   0.138   0.858
E D  4RA0C                115    0.577  83.102  -0.825   0.005   0.336   0.660
B R  4RA0C                116    0.250  57.250   0.562   0.003   0.718   0.279
E T  4RA0C                117    0.523  72.596   0.178   0.001   0.900   0.099
B V  4RA0C                118    0.113  17.383   0.552   0.002   0.816   0.182
E A  4RA0C                119    0.438  48.290   0.871   0.004   0.616   0.381
E A  4RA0C                120    0.481  52.984   0.517   0.004   0.197   0.799
E N  4RA0C                121    0.704 103.095  -0.876   0.005   0.045   0.951
E T  4RA0C                122    0.387  53.705   0.843   0.004   0.085   0.910
E P  4RA0C                123    0.533  75.562   1.439   0.004   0.616   0.381
B F  4RA0C                124    0.043   8.670   0.271   0.001   0.900   0.099
E N  4RA0C                125    0.373  54.563   2.045   0.001   0.959   0.040
B L  4RA0C                126    0.047   8.532   0.822   0.001   0.959   0.040
E S  4RA0C                127    0.338  39.637   1.856   0.001   0.959   0.040
B C  4RA0C                128    0.018   2.457   1.171   0.001   0.959   0.040
E Q  4RA0C                129    0.393  70.136   1.658   0.001   0.959   0.040
B A  4RA0C                130    0.061   6.777  -0.719   0.001   0.900   0.099
E Q  4RA0C                131    0.539  96.265   0.393   0.004   0.616   0.381
E G  4RA0C                132    0.398  31.330  -1.521   0.005   0.262   0.733
E P  4RA0C                133    0.511  72.483   0.032   0.004   0.138   0.858
E P  4RA0C                134    0.252  35.716  -0.634   0.004   0.085   0.910
E E  4RA0C                135    0.582 101.588   0.656   0.004   0.138   0.858
E P  4RA0C                136    0.263  37.263  -0.797   0.004   0.197   0.799
B V  4RA0C                137    0.256  39.301   0.181   0.004   0.420   0.576
E D  4RA0C                138    0.470  67.698   0.916   0.002   0.816   0.182
B L  4RA0C                139    0.047   8.661   0.662   0.001   0.900   0.099
E L  4RA0C                140    0.293  53.667   1.821   0.006   0.962   0.032
B W  4RA0C                141    0.044  10.534   0.398   0.006   0.962   0.032
E L  4RA0C                142    0.262  48.064   1.427   0.001   0.959   0.040
B Q  4RA0C                143    0.214  38.292   1.072   0.003   0.718   0.279
E D  4RA0C                144    0.634  91.302  -0.276   0.018   0.047   0.935
E A  4RA0C                145    0.791  87.179  -1.523   0.005   0.015   0.979
E V  4RA0C                146    0.545  83.720   0.586   0.004   0.616   0.381
E P  4RA0C                147    0.495  70.255   1.062   0.003   0.718   0.279
B L  4RA0C                148    0.046   8.459   0.278   0.021   0.451   0.528
E A  4RA0C                149    0.450  49.546   1.090   0.021   0.279   0.699
E T  4RA0C                150    0.557  77.298  -0.903   0.019   0.141   0.840
E A  4RA0C                151    0.565  62.296  -1.669   0.018   0.047   0.935
E P  4RA0C                152    0.664  94.236  -1.632   0.018   0.047   0.935
E G  4RA0C                153    0.549  43.191  -1.101   0.020   0.205   0.775
E H  4RA0C                154    0.379  68.958  -0.613   0.004   0.420   0.576
E G  4RA0C                155    0.373  29.371  -1.086   0.004   0.420   0.576
E P  4RA0C                156    0.407  57.711  -1.515   0.004   0.420   0.576
E Q  4RA0C                157    0.418  74.726  -0.669   0.004   0.420   0.576
B R  4RA0C                158    0.068  15.503  -0.966   0.004   0.616   0.381
B S  4RA0C                159    0.202  23.651   0.609   0.001   0.900   0.099
B L  4RA0C                160    0.020   3.625   1.076   0.001   0.959   0.040
E H  4RA0C                161    0.335  60.991   1.379   0.001   0.959   0.040
B V  4RA0C                162    0.165  25.422  -0.122   0.001   0.900   0.099
E P  4RA0C                163    0.366  51.978   0.687   0.004   0.616   0.381
E G  4RA0C                164    0.562  44.222   0.565   0.005   0.336   0.660
E L  4RA0C                165    0.269  49.199   0.587   0.004   0.138   0.858
E N  4RA0C                166    0.673  98.556   0.058   0.004   0.085   0.910
B K  4RA0C                167    0.249  51.219  -0.781   0.004   0.085   0.910
E T  4RA0C                168    0.425  58.975  -0.634   0.004   0.138   0.858
B S  4RA0C                169    0.169  19.807  -1.717   0.004   0.616   0.381
E S  4RA0C                170    0.414  48.497   1.623   0.001   0.900   0.099
B F  4RA0C                171    0.054  10.778   0.521   0.001   0.959   0.040
E S  4RA0C                172    0.287  33.601   1.769   0.001   0.959   0.040
B C  4RA0C                173    0.016   2.260   1.408   0.001   0.959   0.040
E E  4RA0C                174    0.275  48.130   2.026   0.001   0.959   0.040
B A  4RA0C                175    0.040   4.419  -0.470   0.001   0.959   0.040
E H  4RA0C                176    0.422  76.798   0.511   0.001   0.900   0.099
B N  4RA0C                177    0.104  15.240  -0.456   0.004   0.514   0.481
E A  4RA0C                178    0.677  74.572   0.317   0.019   0.141   0.840
E K  4RA0C                179    0.394  81.046  -0.509   0.019   0.141   0.840
E G  4RA0C                180    0.445  35.045  -1.093   0.004   0.197   0.799
E V  4RA0C                181    0.527  80.954   0.038   0.022   0.359   0.619
E T  4RA0C                182    0.447  62.041  -0.323   0.004   0.514   0.481
E T  4RA0C                183    0.421  58.379   0.253   0.003   0.718   0.279
B S  4RA0C                184    0.253  29.605  -0.479   0.003   0.718   0.279
E R  4RA0C                185    0.363  83.013   0.352   0.001   0.900   0.099
B T  4RA0C                186    0.301  41.721   0.264   0.001   0.900   0.099
B A  4RA0C                187    0.090   9.951   0.314   0.001   0.959   0.040
E T  4RA0C                188    0.291  40.403   1.161   0.001   0.959   0.040
B I  4RA0C                189    0.075  13.801   0.564   0.000   0.983   0.017
E T  4RA0C                190    0.363  50.306   1.015   0.001   0.959   0.040
B V  4RA0C                191    0.113  17.414   0.532   0.001   0.900   0.099
E L  4RA0C                192    0.364  66.593   0.961   0.004   0.616   0.381
E P  4RA0C                193    0.266  37.731   0.520   0.004   0.085   0.910
E Q  4RA0C                194    0.580 103.659  -0.129   0.018   0.047   0.935
E Q  4RA0C                195    0.817 146.006   0.451   0.000   0.000   0.999

Explain the output.  Go back.
```

---
